# XVII International AIDS Conference: From Evidence to Action - Social, behavioural and economic science and policy and political science

**DOI:** 10.1186/1758-2652-12-S1-S5

**Published:** 2009-10-06

**Authors:** Eric Mykhalovskiy, Glen Brown, Rodney Kort

**Affiliations:** 1 Department of Sociology, York University, Toronto, M3J 1P3, Canada; 2 Glen Brown & Associates, Toronto, M6G 3M1, Canada; 3 Kort Consulting, Toronto, M4Y 2T6, Canada

## Abstract

AIDS 2008 firmly established stigma and discrimination as fundamental priorities in the push for universal access to HIV prevention, treatment, care and support. Conference sessions and discussions reinforced the tangible negative effects of stigma on national legislation and policies. A strong theme throughout the conference was the need to replace prevention interventions that focus exclusively on individual behaviour change or biomedical prevention interventions with "combination prevention" approaches that address both individual and structural factors that increase vulnerability to HIV infection.

Several high-level sessions addressed various aspects of the debate over "vertical" (disease-specific) versus "horizontal" (health systems) funding. The majority of evidence presented at the conference suggests that HIV investments strengthen health systems through the establishment of clinical and laboratory infrastructure, strengthened supply and procurement systems, improvements in health care worker training, and increased community engagement.

Human rights were a focal point at the conference; several presentations emphasized the importance of securing human rights to achieve universal access goals, including workplace discrimination, travel restrictions, gender inequality, and the criminalization of homosexuality, drug use, sex work, and HIV transmission and/or exposure.

## Discussion

### Social, behavioural and economic science

Track D presentations emphasized how social, economic and other contextual processes shape both the HIV epidemic and programme and policy responses to it. To a much greater extent than at previous International AIDS Conferences, HIV-related stigma and discrimination were widely highlighted as primary obstacles to controlling the epidemic. In particular, such discussions explored the intersection of stigma and discrimination with forms of structural inequality based on race, class, gender, age and sexual orientation.

Other AIDS 2008 presentations emphasized the need for "combination prevention", an emerging approach that emphasizes the need to employ multiple, context-specific biomedical, behavioural and structural interventions simultaneously, rather than relying solely on any single intervention. In addition, new research by and for marginalized communities emphasized how progress toward universal access is dependent upon the participation of women, sex workers, IDUs, MSM and other marginalized social groups.

### Prioritizing stigma and discrimination

AIDS 2008 firmly established stigma and discrimination as fundamental priorities in the push for universal access to HIV prevention, treatment, care and support. At a session on evidence-based approaches to stigma and discrimination a strong call was made to elevate the importance of stigma and discrimination reduction in national and international funding, policy development and programming [1]. This call followed the recent publication of a systematic review of scientific research on HIV-related stigma [2]. Mahajan and colleagues note that while HIV-related stigma is widely regarded as a significant barrier to epidemic management, additional research is needed in the areas of defining and measuring stigma, understanding the relationship between stigma and HIV testing and treatment rollout, and the impact of stigma-reducing initiatives. These findings are consistent with the *2008 Global Report on AIDS*, which stressed the extent to which stigma, discrimination and human rights violations were impeding progress on universal access targets [3].

**Figure 1:**
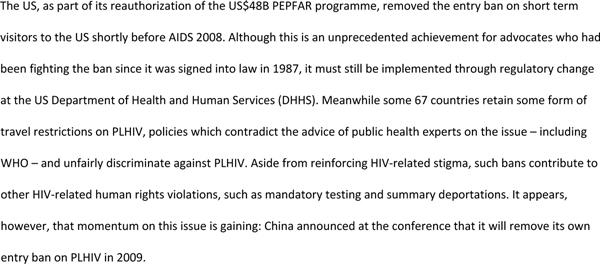
**Travel restrictions for PLHIV**.

Although references to stigma proliferated at the conference, there is often little attempt to conceptualize what is meant by that term, particularly in the context of social science research which attempts to identify and evaluate, through scientific inquiry, its forms, impacts and potential remedies. Two important directions for how stigma is conceptualized were suggested by multiple presentations. The first is to abandon the pursuit of either individual or structural level approaches to stigma reduction in favour of a conceptual approach emphasizing their interconnectedness [4-8]. The second is to approach HIV-related stigma as a layered phenomenon fundamentally linked with and enabled by sexism, racism, homophobia, the stigmatization of IDUs and sex workers, and other forms of social inequality [9-13]. Further developing these key approaches to stigma through conceptually-focused work and developing ways to operationalize them are important objectives for social science research in the area. One of the more tangible structural implications of stigma and discrimination are the travel restrictions imposed by many countries on PLHIV (see Travel Restrictions sidebar). Many countries commit rhetorically to addressing stigma and discrimination faced by people living with HIV while ignoring some of the most obvious and tangible legislative and policy demonstrations of such stigma, such as travel restrictions aimed at PLHIV (see Figure 1).

At the conference, important progress was reported in developing standardized tools for assessing levels of stigma. Research from Puerto Rico and India described validation processes for stigma indices involving PLHIV and rural men, respectively [14,15]. While these instruments focus on sociocognitive dimensions of stigma, another exciting initiative designed to assess stigma at both the individual and structural levels - the People Living with HIV Stigma Index - was reported by Yuvaraj and colleagues [16,17]. The PLHIV Stigma Index is intended to measure change in stigma over time and to allow for country comparisons to inform programme and policy interventions, as well as advocacy. The index combines research with empowerment by placing PLHIVs at the centre of the process as interviewers, interviewees and as local users of the information generated. The lessons learned from the global roll-out of the instrument, currently underway, will help clarify the potential of this unique community-centred approach to stigma surveillance and response.

Research presented at AIDS 2008 also highlighted the complex relationship between stigma and access to prevention, treatment, care and support. Two key debates in the field were the focus of considerable discussion: the relationship between HIV-related stigma and routine (provider-initiated or "opt-out") HIV testing and the extent to which broader access to ART affects the level of HIV-related stigma. With regard to routine testing, several studies pointed to the need to harmonize tensions between population-based and rights-based approaches to HIV testing, including how to interpret research results [18-23]. Two additional studies on whether treatment rollout itself contributes to stigma reduction yielded contradictory results, with one pointing to increased perceived stigma with treatment use and the other suggesting a decline in stigmatizing attitudes with increased availability and access to ART [24,25]. The contradictory nature of these studies points to the need for future research that can more effectively identify the mix of individual and structural conditions that mediate the relationship between HIV-related stigma and treatment uptake.

AIDS 2008 also featured research on HIV-related stigma among health care workers. Pilot testing of a stigma reduction programme conducted in Yunnan Province, China showed high levels of fear of infection among health care workers and little knowledge of universal precautions [26]. Following the intervention, an improvement was observed in maintaining the confidentiality of HIV-positive clients and for voluntary counselling and testing (VCT). A second study in Vietnam assessed the outcomes of a community-led stigma reduction campaign [27]. Results at 16-months following baseline demonstrated a positive relationship between exposure to the number of campaign initiatives (e.g. community education, billboards, fact sheets) and reductions in stigma scores and fear of transmission. By focusing on the health care sector, these two initiatives help assess structural dimensions of stigma. They provide an important example for future research targeting other structural sources of stigma, including government policy, religious institutions, and criminal justice systems.

### New consensus: "combination HIV prevention"

AIDS 2008 underscored a new direction in HIV prevention. In place of prevention interventions focused exclusively on individual behaviour change, numerous speakers called for "combination HIV prevention", which encourages a more long-term approach to reducing HIV risk and vulnerability by addressing both individual and contextual factors [28]. Combination prevention draws on multiple risk reduction strategies rather than relying upon a single "magic bullet", and takes into consideration the relationship between prevention programming and politics, particularly at the level of community involvement and activism [29]. Research and commentary at AIDS 2008 contributed to this new direction in HIV prevention in two important ways.

First, analyses of the political, social, and cultural implications of specific prevention strategies helped temper widespread assumptions about their universal application. For example, in a session on male circumcision, speakers argued that insufficient attention is given to anthropological research on the body, culture and on masculinity by those considering the impact of scaling up this intervention [30]. Similarly, Beloqui (Associação Brasileira Interdisciplinar de AIDS, Brazil) noted the limited reach of HIV prevention efforts targeting individuals who are HIV-positive (also referred to as positive prevention) in the Brazilian context, where most PLHIV do not know their HIV status. Beloqui suggested that when restricted to the level of individual behaviour change, positive prevention can have the effect of "blaming" PLHIV for transmission, while also eroding traditions of mutual responsibility for HIV prevention. He argued instead for a vision of positive prevention that enhances the social capital of PLHIV, as well as their access to treatment and prevention technologies, while also working at the policy level to reduce stigma and enhance human rights [31].

Second, AIDS 2008 generated considerable interest in structural forms of HIV prevention and their relationship to behavioural and biomedical prevention strategies. Research on a range of structural interventions was presented at the conference, including efforts to reform policing practices affecting IDUs, to introduce micro-financing initiatives for at risk women, and to enhance community mobilization of sex workers [32-34]. In a session on rethinking the role of structural issues in HIV prevention, several analyses of innovative developments in prevention programming were offered [35]. Birungi described a Kenyan initiative that seeks to shift the organization of HIV programming. In a context where the response to the epidemic has been built primarily around paediatric and adult HIV care and prevention, Birungi (Population Council, Kenya) and colleagues turned their attention to the relatively neglected population of HIV-positive youth. Rather than seeking individual behaviour change, they have worked to better understand the gendered construction of sexuality among HIV-positive youth, addressing research questions about their sexual desires, relationship expectations and hopes for love and parenthood in an effort to establish a knowledge base on which to build relevant interventions.

The most significant impact of social science research on combination prevention likely lies ahead. Future research might turn to the strengths of contemporary sociological theory on the relationship between structure and agency to deepen our understanding of the concept of structure as it is deployed in the emerging discourse on combination prevention [36,37]. At the same time, social science approaches such as social networking, actor network theory, and the analysis of causal pathways have much to offer in better understanding how structures relate one with another and how structural interventions interact with biomedical and behavioural interventions. Finally, as Ogden noted, methodological innovation in the social sciences is needed to understand whether and how structural HIV prevention interventions reduce HIV transmission [38].

### Research on, for and by marginalized communities

Another important development at AIDS 2008 was the expansion in HIV research involving vulnerable and marginalized communities, particularly in contexts where sex work, injecting drug use, and homosexuality are criminalized and/or are not officially recognized as significant issues for HIV prevention. Critical analyses and commentaries articulating the perspectives of marginalized communities offered important insight into the political, economic and social conditions that heighten HIV vulnerability for MSM, sex workers and IDUs, while also shedding light on efforts to change those conditions. Within Track D, particular attention was paid to MSM and male and transgender sex workers [39,40].

In his Jonathan Mann Memorial Lecture, Jorge Saavedra, Director of Mexico's National Centre for HIV/AIDS Prevention and Control (CENSIDA) presented an overview of research demonstrating that HIV prevalence among MSM is significantly higher than in the general population, even in Africa and other areas with generalized epidemics. He presented other studies to illustrate the relative paucity of resources dedicated to MSM prevention. Saavedra argued forcefully that homophobia - from common forms of discrimination to more extreme forms such as laws criminalizing sex between men - fuels the spread of HIV [41]. He also called on Ugandan authorities to drop the charges against three gay and lesbian rights activists who were arrested at the June 2008 HIV/AIDS Implementers Meeting in Kampala (one of whom was tortured); on August 15 all charges against the three were dropped and the regional chair of the International Gay and Lesbian Human Rights Commission (IGLHRC) attributed it directly to advocacy at AIDS 2008 [42].

Saavedra's plenary presentation built on a two-day pre-conference on issues facing gay and other MSM in the global response to the epidemic. One of the sessions at the pre-conference outlined an innovative approach to forging government support among countries in the Greater Mekong Sub-region (Thailand, Vietnam, Cambodia, Laos, Myanmar and two southern provinces of China). The programme, which involved compromises in the language used with government officials (e.g., use of "male sexual health" rather than "MSM" in initial meetings), strong local coordination and support, and a coalition between government, civil society and donors resulted in fundamental policy shifts within governments, and the inclusion of MSM as a priority within all national AIDS plans in the Greater Mekong Sub-region [43]. Similar successes were reported by Shivinanda Khan (Naz Foundation International/APCOM, India), who outlined an iterative process involving a series of meetings - including informal park gatherings - that resulted in growing support among MSM, government and civil society groups that led to hosting the Asia-Pacific Consultation on Male Sexual Health in September 2006 [44]. These examples, while critical, also serve to underscore the structural barriers that MSM communities and networks face in developing an appropriate policy or programmatic response.

**Figure 2:**
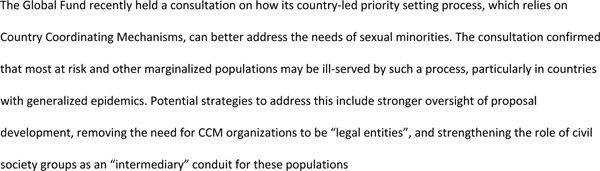
**Sexual minorities and global fund CCMs**.

The title of the MSM pre-conference, The Invisible Men: Gay Men and Other MSM in the Global HIV/AIDS Epidemic, highlighted the notion of invisibility, which often characterizes these communities, particularly in generalized and low-level epidemics. Research conducted in Togo illustrated the challenge of investigating same-sex HIV issues in climates of invisibility. The research team was told by Togo government officials not to bother with their proposal to research MSM because they did not exist. The team persisted with a community-based ethnographic approach that identified 122 MSM and discovered key gaps in their knowledge of HIV risk. One of the results of the research project was the development of new MSM networks and programmes [45]. An example of how to address the issues faced by sexual minorities through national AIDS funding mechanisms is addressed in Figure 2.

Reports from another popular session on MSM issues at AIDS 2008 yielded positive evaluation data on: an Australian five minute, five question assessment tool to screen for likely sexual risk behaviour [46]; use of a mobile van to provide HIV tests for hard-to-reach Peruvian MSM [47]; and an interactive internet quiz to help French and Dutch gay men identify their triggers for non-premeditated risk taking [48].

In an abstract driven session on male and transgender sex workers held at an International AIDS Conference, pioneering research was presented on the HIV risks faced by these two populations and on important initiatives designed to intervene in conditions that heighten their risk. Hunter (Thailand) underscored the invisibility of such groups at all levels of the HIV response [49]. He noted that few national HIV/AIDS programmes explicitly target these groups, and argued that subsuming programmes for transgendered sex workers in MSM funding streams has created obstacles for HIV programming that responds to the particular needs of these sex workers.

Suggesting a way forward, research from Thailand and Peru offered examples of early efforts to establish an evidence base from which to inform targeted programming that better responds to the HIV risks faced by transgender and male sex workers [50,51]. Other research profiled interventions designed to alleviate structural factors that heighten HIV risk. In Columbia, transgender sex workers often choose not to carry a national identification card - a legal requirement and a condition for receiving health care - because of the mismatch between biological sex at birth and later gender identity. Riascos Sanchez (Santamaria Fundacion GLTB, Columbia) described a project initiated by Fundación Santamaría, a community-based organization of transgender people that responds to the vulnerabilities faced by this population [52]. By providing an alternative identification card that includes the bearer's chosen name, and by engaging in training and advocacy with the police and health care providers, the project has enhanced transgender sex workers' access to health services and contributed to decreased police harassment.

The research on, for and by marginalized communities presented at AIDS 2008 will have important implications for future policy and research. At the epidemiological level, a refinement of the concept of "generalized epidemics", or at least a greater understanding of its potentially exclusionary effect, was strongly suggested by research pointing to the high burden of HIV among MSM in countries designated as having generalized epidemics. The conference's increased focus on the role of structural factors that contribute to HIV risk and vulnerability also underscored the need for global advocacy on such issues, and for additional social science research and new analyses that are able to capture the complex interaction between individual risk behaviour and social vulnerabilities. Finally, the opportunity for community activists and members of marginalized groups to present their research in an international forum, and to be able to discuss how to use such evidence to inform global advocacy, will continue to have important implications for the regional and international mobilization of MSM, IDUs and sex workers.

### The role of social science research and acceptable evidence

AIDS 2008 included a number of important debates about the role and presence of the social sciences in HIV/AIDS research, and the related question of what types of scientific research evidence are privileged in responding to the epidemic. Given the dominance of biomedical and health sciences, the social sciences are often viewed as junior partners in HIV/AIDS research. Although the conference dedicates a separate track to the social and behavioural sciences, the actual social science content of presentations in the track is not always apparent, although this may be a reflection of less robust investments in social science compared to biomedical research in the HIV field. While presentations referenced social science disciplines (such as sociology), the full range of social sciences was not well-represented at the conference, with contributions from history, geography, economics and anthropology most obviously absent. Attracting relevant social science research and realizing its potential in the response to the epidemic remains an ongoing challenge.

Debates about what are considered acceptable forms of evidence and alternative ways of producing useful knowledge that can be used to move us closer to universal access occurred in a number of sessions at the conference [52-57]. In a symposium on the current scientific and political challenges of evidence-based HIV prevention sponsored by the Caucus for Evidence-based Prevention, presenters questioned the use of the randomized control trial (RCT) as the accepted gold standard for research evidence [58]. Judith Auerbach (San Francisco AIDS Foundation, USA) noted that the RCT is not well-suited to addressing the interplay of physiological, social, cultural and structural factors that is increasingly recognized as fundamental to HIV prevention. Rafael Diaz (San Francisco State University, USA) further argued that the institutionalization of the RCT as a funding criterion for HIV prevention, particularly in the US context, has resulted in fidelity to a "standardized protocol", which creates obstacles for creative prevention research that begins with local communities in a "bottom up" rather than "top down" fashion [59]. The session made a strong case that exploring the significance of poverty, gender, economic inequality, racism and other structural relations for HIV prevention initiatives will require moving beyond the RCT and traditional research methods to include a range of social science disciplines, innovative evaluation designs, and community-based research.

### Policy and political sciences

Track E featured new research and commentary on key barriers to universal access to HIV prevention, treatment, care and support. In multiple presentations, speakers reiterated the growing concern that international and national financial commitments are falling short of what is needed to deliver on universal access goals [60-62]. In addition, the conference highlighted key findings and research on human rights and health systems strengthening, and has contributed to more widespread awareness of the fundamental importance of these issues for achieving universal access.

AIDS 2008 brought to the forefront the importance of a health and human rights-based approach to HIV. Human rights were a focal point of many activities in Mexico City, including: marches on homophobia, women's rights, and housing; the first ever Global Village "Human Rights Networking Zone"; and the circulation of key UNAIDS and Open Society Institute publications on human rights and HIV [63-65]. The importance of securing human rights as a prerequisite to achieving progress towards universal access was emphasized through new research on topics such as workplace discrimination, travel restrictions, and the denial of women's property and inheritance rights [66-68].

Key themes emerging from AIDS 2008 addressed the human rights context of drug use and sex work, the criminalization of HIV transmission/exposure, and the challenge of incorporating human-rights principles in HIV programming. The contentious issue of patent protection and drug pricing, including a new initiative that is focused on bridging differences between stakeholders on this topic, was also among prominent issues of discussion.

### Injection drug use, sex work and human rights

AIDS 2008 featured important work documenting the magnitude and nature of human rights abuses faced by drug users and sex workers. It also explored the institutional sources of such rights abuses and highlighted key areas of advocacy and policy reform. Suwannawong (Thai AIDS Treatment Action Group, Thailand) reported on field research conducted in five Thai provinces demonstrating that drug users are systematically denied access to ART and HIV/AIDS information, and routinely face police harassment [69].

Other research presented by plenary speakers pointed to regular police harassment of clients and workers at needle exchange programs, even in contexts where such services are legal [70]. In the first plenary address on sex work given by a sex worker at an International AIDS Conference, Elena Reynaga (RedTraSex Latinoamérica y el Caribe, Argentina) gave a powerful account of human rights abuses faced by sex workers, including mandatory testing, denial of health services and coercive health interventions [71]. Results of participatory action research conducted by sex workers in twelve countries and presented at the conference by Anna-Louise Crago (Sex Workers' Advocacy Network of CEE/CA, Hungary) reinforced Reynaga's claim. The study documented widespread police harassment of sex workers, with 41% of participants reporting physical violence by police in the past year [72].

Multiple presentations at the conference drew attention to how public health and human rights-based approaches to sex work and drug use have been displaced by strategies emphasizing criminalization and law enforcement [73-75]. This has fuelled problematic police practices such as confiscation of needles obtained from syringe programmes, police raids and violence against sex workers. Police often treat condoms in a woman's possession as evidence of prostitution thereby discouraging condom use. Such practices place drug users and sex workers at elevated risk for HIV transmission. Research indicated that a "law and order" response to sex work and drug use leads to laws, regulations and policies that hinder access to HIV services for sex workers, injection drug users and other marginalized populations in 63% of countries that submitted UNGASS progress reports [76]. Such evidence highlights the growing tension in the policy arena between human-rights based approaches to health and criminal law/policing strategies. In response, numerous speakers at AIDS 2008 articulated a strong call to decriminalize sex work and drug use, and to remove all legal restrictions to harm reduction services.

A session on policy responses to IDUs illustrated the challenge of collecting meaningful data on injecting drug use and HIV that can be used to affect public policy. The Reference Group to the UN on HIV and Injecting Drug Use presented the results of recent efforts to assemble global data on the prevalence of injecting drug use and of HIV prevalence within IDU populations. The reference group was able to conduct a detailed analysis of 3,000 peer-reviewed and grey literature, from an initial total of 15,000 [77]. The reference group concluded that injection drug use is well-established in many regions (with particularly high rates of injecting drug use in Russia, China and the US), and that HIV prevalence among IDUs is high and growing: between 700,000 and 6.6 million of the 11 million to 21.2 million IDUs globally are HIV-positive.

The data on HIV prevalence among IDUs and evidence-based interventions for this population are only sporadically reflected in national public policy responses to drug use. Human Rights Watch reported on efforts to influence drug policy in the Russian Federation. Russia's policies - from criminalizing drug use, to banning substitution therapy and providing dangerously ineffective treatment options - contradict scientific evidence, and Russian authorities are openly hostile to dissent on the topic [78]. The recently-formed International Network of Drug Consumption Rooms reported that, despite considerable evidence of their positive impact, consumption rooms exist in only eight countries and, as Canada's Minister of Health demonstrated in his forceful opposition to such facilities at the conference, even these are subject to intense political opposition [79]. A review of evaluations of prison needle exchange programs (PNEPs), which currently operate in 60 prisons in 11 countries found that needle sharing was sharply reduced and that there were no cases where a needle had been used as a weapon, a frequently cited concern of corrections staff [80]. The evaluations also demonstrate improved health outcomes and disprove fears that PNEPs lead to increased drug use. Despite this evidence, governments continue to resist expanding the number or scope of PNEPs [81].

Research presented in Track E also described how international policy can exacerbate the human rights context of drug use and sex work. One particular set of concerns focused on the resulting confusion and lack of direction stemming from contradictory and inconsistent international policies. Mina Seshu (Sampada Grameen Mahila Sanstha (SANGRAM), India) noted that, while UNAIDS formally recognizes sex workers as key partners in policy formation, its own 2006 *Guidance Note on Sex Work *ignored sex worker input. Rather than stressing the need to decriminalize sex work and emphasizing that HIV incidence among sex workers is declining, the report instead focused on eliminating sex work altogether [82]. Another concern was raised about funding structures that marginalize sexual and reproductive rights. For example, drawing on interviews and policy research, Sippel (Center for Health and Gender Equity, USA) and colleagues reported that PEPFAR's anti-prostitution policies and ideological commitment to abstinence and "faithfulness" have complicated local efforts to respond to gender-based violence, and have impeded comprehensive sexual and reproductive health services for marginalized PLHIV, including women, immigrants and sex workers [83,84]. The reform of PEPFAR funding priorities and restrictions was identified as a critical area of policy advocacy in the future.

### Criminalization of HIV transmission

AIDS 2008 established the use of criminal laws to prosecute PLHIV who transmit HIV or expose others to HIV infection as one of the most pressing issues facing the global AIDS movement. Presentations coalesced around the argument that criminalization of HIV transmission is bad public policy, and emphasized that there is a lack of evidence demonstrating that the application of criminal law will prevent HIV transmission, and the very real possibility that it will heighten stigma and discrimination. Justice Edwin Cameron's (Supreme Court of Appeal, South Africa) plenary presentation on the issue provided an incisive and sobering global overview of the growing trend towards criminalizing HIV transmission, even in cases where transmission does not occur, and the multiple ways in which this undermines an effective response to HIV [85].

New research contributed to the understanding of the issue of criminalization of HIV transmission in three central ways. First, Pearshouse (Canadian HIV/AIDS Legal Network, Canada) presented monitoring data on the uptake of criminal laws for HIV transmission/exposure at the global level and offered an incisive critique of the USAID-sponsored Model Law process [86]. He argued that the process has produced a rash of HIV-specific laws in Western Africa, many of which exceed the provisions of the Model Law itself. Recently passed laws include problematic restrictions on youth-directed HIV education and overly broad provisions for criminalizing HIV transmission and exposure that in some instances extend to mother-to-child transmission. Several presentations at AIDS 2008 challenged the merits of the Model Law process and other initiatives that heighten criminalization of HIV transmission/exposure. Rather than proliferating HIV-specific laws with a dubious relationship to HIV prevention, various presenters argued that criminal law be applied only to cases of intentional and actual HIV transmission, and that law reform be redirected to address key drivers of HIV transmission, including violence against women and the criminalization of same-sex relationships [87-90].

**Figure 3:**
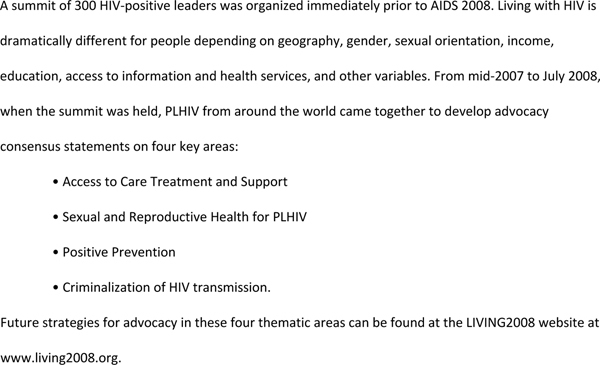
**LIVING 2008 positive leadership summit**.

Second, serious questions were raised about the presumed protective value for women of criminal laws against HIV transmission/exposure. In sub-Saharan and East Africa, much of the impetus for criminalizing HIV transmission has come from women's organizations trying to protect women from infection through the statutes. Clayton and Gathumbi (Namibia) argued that newly generated criminal laws do not realize such good intentions but, instead, threaten women's health and human rights [91,92]. They noted, for example, that criminal prosecutions will likely fall disproportionately on women, who are often the first in heterosexual relationships to learn of their HIV status in antenatal clinics, and who are often blamed for "bringing HIV into the home" [93]. Due to the threat of violence and mistreatment, many women are disinclined to disclose their HIV status. Rather than protecting women, the broad disclosure requirements in some criminal statutes - for example the current Tanzanian requirement for immediate disclosure to a spouse or sexual partner - places them at risk for criminal prosecution [94]. A two-day pre-conference Positive Leadership Summit included criminalization of HIV transmission as one of four key global advocacy issues for people living with HIV (Figure 3).

Finally, an important discussion arose during AIDS 2008 about the state of research on criminalization of HIV transmission/exposure. Most new research presented at the conference took the form of surveys of national developments related to criminalization or human rights analyses of legal texts and processes. To further arguments against the criminalization of HIV, additional research that explores the claims that such laws: (1) increase HIV-related stigma; (2) deter people from testing for HIV infection; and (3) promote a false sense of security amongst those who are uninfected is needed [95-99]. Progress towards these research questions will be much anticipated at AIDS 2010.

### Moving beyond rhetoric

Adopting a health and human rights framework for responding to the HIV pandemic involves more than rhetorically embracing human rights principles; it involves operationalizing such principles in the delivery and assessment of HIV services [100]. Research presented at AIDS 2008 showed that there has been mixed results in this regard. On one hand, research presented at the conference identified a range of rights-based programmes currently in use. For example, Patel (Southern Africa Litigation Centre, South Africa) described an ambitious attempt in Africa to intervene in legal proceedings using an impact litigation framework [101]. Established in 2007, the Strategic Litigation Fund, which operates everywhere in Southern Africa except South Africa, seeks to strengthen human rights by providing monetary and technical assistance to lawyers, and by mobilizing communities around human rights issues. Thus far the Fund has successfully intervened in cases dealing with issues such as inequity in women's property rights and discrimination against people living with HIV in the military.

**Figure 4:**
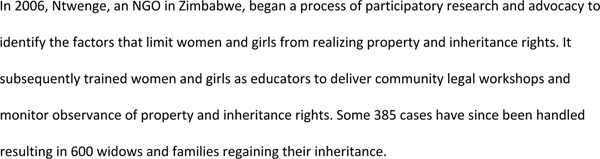
**The Ntwenge initiative**.

Markham Ntwenge, an NGO in Zimbabwe that engages in programmes related to the health and economic rights of women, girls and orphans, provides another interesting example (see Figure 4) [102]. Mary Robinson (Ireland) identified gender inequality and violence against women - particularly the devastating impact of rape and other forms of assault on women living in conflict settings - as a human security issue with profound implications for women's access to HIV and other health services [103].

While there are promising examples of tangible programmes, research presented at AIDS 2008 demonstrated that operationalizing human rights-based HIV programming remains underdeveloped. The conference made important, if preliminary, progress toward that end on two fronts. First, Gruskin (Harvard School of Public Health) and colleagues pushed the discussion of what a human rights-based approach actually means in practice by developing an operationalization framework for use at the country level that includes items about how key human rights principles have been integrated in the design, delivery and evaluation of programmes [104]. Second, Ferguson (Harvard School of Public Health, USA) and colleagues discussed how to define suitable indicators for use in formally evaluating rights-based approaches [105]. Their research emphasized the utility of drawing on the publicly available National Composite Policy Index, which includes indicators that capture information on key human rights principles such as non-discrimination, participation and accountability.

### Access, drug costs and patents

A number of studies and presentations examined the complex issue of the cost of pharmaceutical medicines as it affects access to treatment. Presenters described a variety of approaches that have significantly reduced the price of treatments in most developing countries. Many challenges and debates remain on this highly contested terrain, especially as the need for second-line therapies increases and the demand for simplified or fixed drug formulations, pediatric formulations and therapies to address TB escalates.

A session on "Universal Access, Universal Crises and Universal Prices" presented specific approaches and challenges to the access and patent issue. A Brazilian report described that country's experience in financing universal ARV access by applying strategic pressure on the industry - negotiating for the lowest possible brand-name price in some instances, and importing or producing generics in others. The report noted that the increasing need for and cost of second line therapies is straining the country's resources [106]. A Canadian report illustrated the limitations of "Canada's Access to Medicines Regime" legislation, which is intended to provide generic products for export to low income countries. Elements of the legislation and its regulations - such as regulations requiring a review of the requested generic import by Canada's Therapeutic Products Directorate after the manufacturer and requesting country have already reached agreement on price and quantity - produce significant barriers to achieving its goals, to the extent that so far only one drug has been approved for export [107].

Médecins Sans Frontières (MSF) described the successes, failures and frustrations of the "hand-to-hand combat" approach - strategic battles on each drug and each combination in each country. MSF and other NGOs are now proposing a model called "patent pooling" whereby pharmaceutical companies would, in return for a negotiated royalty, allow access to their patent formulas for public suppliers and generic manufacturers to supply lower cost drugs to low-income nations. According to MSF the concept is receiving positive responses from some donors, governments and pharmaceutical companies and momentum appears to be growing in support of this initiative [108].

### Global HIV/AIDS initiatives and health systems strengthening

**Figure 5:**
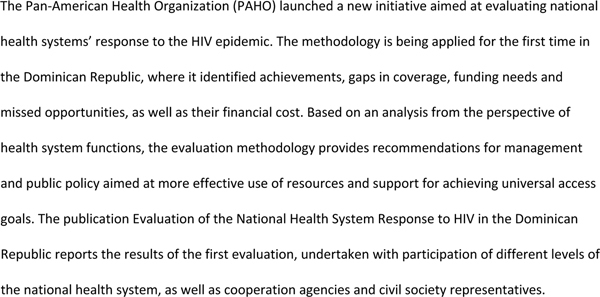
**Evaluating health systems for their HIV response**.

AIDS 2008 firmly established the intersection between HIV-specific global programmes and sector-wide health care provision and reform as a fundamental issue for the global HIV/AIDS movement. The attention paid to the issue at the conference followed the publication of articles claiming that HIV-specific global initiatives are over-resourced compared to other health issues, poorly integrated with general health systems, cost-ineffective and that vertical funding for AIDS distorts global health priorities [109-111]. A number of speakers challenged the underlying rationale of this debate, arguing that scaling up and sustaining universal access to HIV prevention, treatment, care and support will only be achieved by substantially investing in and strengthening comprehensive primary care and general health systems [112-114]. This position was supported and elaborated in three important ways. First, the polarized and polemic nature of the debate was reframed. Second, important research on the effects of HIV-specific funding on global health systems was presented. Finally, speakers identified key directions for future research and programmatic responses to this issue (see Figure 5).

### Reframing the health systems debate

At AIDS 2008 numerous speakers challenged the false dichotomy between HIV programmes and global health services. Tedros Ghebeysus, Julio Frenk and others challenged this framework at the most basic level, noting that HIV services are themselves health services that do not exist independently of a general health system [115,116]. Frenk traced the roots of this dichotomy to the distinction between a health system with explicit priorities, termed a vertical approach, and a horizontal approach, which "strengthens the health system in general, but without priorities." He cautioned against the latter, noting that horizontal approaches typically are most beneficial for the most affluent members of society.

Multiple presenters argued that framing the debate as one of HIV-specific services versus the general health system is an outmoded and overly simplistic approach [117-119]. Instead, a diagonal strategy was recommended in which targeted improvements in prioritized services generate spill-over effects to other areas of health services delivery. According to Frenk, "diagonal thinking" shifts the debate from an oversimplified dyad to the question of which explicit mix of prioritized services are most appropriate, and what degree of integration among them is needed to form a unified health system suitable for a given context.

A related effort to reframe the debate focused on claims that HIV-specific funding initiatives and the successful lobbying efforts of international AIDS activists have damaged global health by preventing the development of robust primary health services in developing countries. In a compelling critique of this argument, Gregg Gonsalves (AIDS and Rights Alliance for Southern Africa/International Treatment Preparedness Coalition, South Africa) noted that, while HIV/AIDS has exposed health systems weaknesses, those weaknesses predated the emergence of HIV/AIDS as a global health problem [120]. Rather than blaming AIDS activists, a more historically accurate argument would draw attention to the structural adjustment policies of international financial institutions that drained resources from public health and welfare systems in developing countries. Gonsalves and other presenters further noted that the HIV response has, in fact, revitalized global public health, helping to focus attention on the problem of underfunded health services in low-income countries while fostering strong civil society demands for greater involvement in improved health services generally [121,122].

### The impact of HIV-specific initiatives

**Figure 6:**
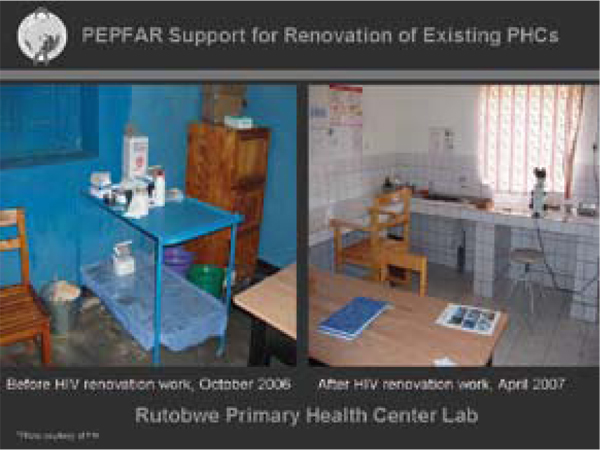
**PEPFAR support for renovation of existing PHCs**. Source: Dybul, M. Human capacity development in the US, President Emergency Plan for AIDS Relief (MOSAT1605).

Presenters at AIDS 2008 also urged a more nuanced consideration of the impact of HIV-specific programmes on overall health systems. Much of the discussion focused on demonstrating how "vertical" HIV services have generalizing effects (Figure 6). For example, Michel Kazatchkine, Executive Director of the Global Fund, argued that HIV-specific funding has a significant impact on the burden of disease, one important effect of which is to free up hospitals from the pressures of caring for HIV-positive patients and "allowing hospitals to become hospitals again" [123]. Others noted that HIV-specific health funds help to train and support health workers, build infrastructure for the delivery of primary health care, develop procurement and distribution systems for a wide variety of medications, and strengthen health care monitoring and evaluation (Figure 6) [124].

At the same time, it was acknowledged that HIV-specific initiatives can complicate health systems integration. In some countries HIV-specific funding has strained government/NGO relationships and has not been sufficiently integrated with public health systems, poverty reduction strategies and other efforts to build human resource capacity and develop infrastructure [125-127]. A study of six African countries documented an explosion of new organizations responding to HIV, most with little infrastructure and short-term projects that provide little in the way of a sustainable programme [128]. A study of Global Health Initiative (GHI) funding in Uganda found virtually no coordination or alignment between funders and in-country coordination mechanisms [129], and a separate review of GHI programmes in Malawi and Zimbabwe noted that these new resources had little positive impact on health care worker shortages [130]. Another report showed that one small country, Kyrgyzstan, had funds flowing in from no less than 17 different international funding bodies, with different application and reporting mechanisms [131]. In another study on the proliferation of independent funding streams, Mphu Ramatlapeng and Ghebeysus noted that different financial, reporting and accountability procedures across donor agencies create problems of harmonization at country levels [132,133]. Drawing on this critique, the authors emphasized the importance of moving quickly to implement the recommendations of the Paris Declaration on AID Effectiveness.

### Moving forward on a shared agenda

AIDS 2008 laid the foundation for moving forward on a revitalized approach to the relationship between HIV-specific initiatives and other health services. At the programmatic level, considerable attention was paid to measures with the potential to enhance access to HIV prevention, treatment, care and support, while also strengthening primary health care capacity. Addressing the widespread shortage of health-related human resources in low-income countries was a particularly prominent theme. A number of presentations emphasized the need to enhance donor support for health workforce training and compensation, while also responding to the ongoing drain of professional health workers due to burnout, migration and the movement out of the public sector to NGOs, faith-based organizations and the private sector [134-136]. Other presenters noted the positive impacts of providing treatment to HIV-positive health care workers to keep them in the workforce [137]. Decentralizing testing and care, in an effort to bring primary care to the front-lines, was advocated by several presenters [138].

Two systematic reviews of the literature presented at AIDS 2008 showed mixed results on the health system effects of global HIV/AIDS initiatives. Atun noted that a recent Cochrane review provided limited evidence either for or against general health system effects, while a review of the evidence in seven countries presented by Rao showed positive health system effects in civil society decision-making and in national decentralization initiatives [139,140]. Both reviews suggest the need for additional empirical research that will provide an evidence-base for answering the question of how best to enact the integration of disease-specific health priorities in given contexts. Kim (Francois Xavier Bagnoud Centre for Health and Human Rights, USA) described an emerging research programme that seeks to do just that [141]. It involves a multi-institution partnership that will draw on new forms of expertise, including systems engineering and operations research, qualitative and quantitative data, theory building and innovative methodologies of assessment to comparatively explore how global health initiative investments are used. The goal is to identify the context-specific and context-independent principles behind successful integration that might help guide future health systems decision-making.

## Conclusion

Approaches for conceptualizing, implementing and evaluating interventions aimed at reducing HIV-related stigma, such as the PLHIV Stigma Index, brought much-needed methodological rigour and focus to this issue, emphasizing the multiple effects of HIV stigma and other structural barriers on access to prevention, care and treatment services. Discussions and presentations also reflected the growing momentum of "combination prevention' as an approach which recognizes the need to deploy multiple evidence-based interventions that address both individual and structural barriers to reducing the spread of HIV (such as microfinancing initiatives aimed at increasing the economic independence of women and girls).

An increasingly robust body of research by and for marginalized communities described contexts where sex work, injecting drug use, and homosexuality are criminalized and suggested avenues for moving forward on an advocacy agenda with political leaders to deal with structural barriers to access in the context of challenging social, legal and political terrain. Concerns about the increasing 'biomedicalization' of scientific knowledge and AIDS discourse over the past several years, including how the randomized control trial - the 'gold standard' of clinical scientific inquiry - has eclipsed other mechanisms of knowledge production, will hopefully be addressed in future conferences, where social sciences could provide important analyses and evidence for understanding the complex dynamics of the epidemic and how best to deliver HIV and related health services.

Track E sessions provided tangible examples of how to move beyond the rhetoric of a human rights-based approach on health and HIV to implementing and evaluating initiatives that incorporate human rights principles as core components of service delivery and community-based advocacy. Compelling critiques of how the increasing trend towards criminalizing HIV exposure and transmission undermines an effective public health response to the epidemic will need to be supported by research which explores the impact of such laws on HIV-related stigma, the uptake of testing and counselling services, and individual sexual practice.

The patent pooling concept proposed by UNITAID as a possible mechanism to facilitate ART access while avoiding the internecine battles among governments, NGOs and innovator pharmaceutical companies, will be important to follow, particularly as increasing numbers of PLHIV in resource-limited settings require access to more expensive second and third-line drug regimens.

Additional research is required to supplement existing studies demonstrating the tangible benefits of HIV investments to the health care system (such as laboratory infrastructure, health workforce training and community involvement); perhaps equally important are related calls to minimize health system distortions and maximize coordination among donors by implementing the Paris Declaration on Aid Effectiveness. A convincing critique of the assumptions underlying the notion that HIV investments were undermining primary health care and other health priorities was delivered by several presenters, and has had the salutary effect of strengthening the drive to better integrate HIV with other health services. The research programme currently in development at the François-Xavier Bagnoud Center for Health and Human Rights could provide important new insights into the most effective strategies for delivering health interventions within a social justice framework.

## Competing interests

Eric Mykhalovskiy, Glen Brown and Rodney Kort are independent consultants contracted by the International AIDS Society for the purpose of preparing and editing the AIDS 2008 Impact Report for publication.

## Authors' contributions

Eric Mykhalovskiy and Glen Brown drafted the initial text and Rodney Kort provided editorial input and advice. All three authors have approved the manuscript for publication.
